# Performance Comparison of Polymeric and Silica-Based Multi-Bed Pervaporation Membrane Reactors during Ethyl Levulinate Production

**DOI:** 10.3390/membranes12101000

**Published:** 2022-10-14

**Authors:** Kamran Ghasemzadeh, Milad Ghahremani, Elham Jalilnejad, Taher Yousefi Amiri, Angelo Basile

**Affiliations:** 1 Faculty of Chemical Engineering, Urmia University of Technology, Urmia 57166-93187, Iran; 2 Chemical Engineering Department, University of Zanjan, Zanjan 45371-38791, Iran; 3 Hydrogenia S.r.l., Via Roma, n. 8/2, 16121 Genoa, Italy; 4 Department of Engineering, University Campus Bio-Medico of Rome, Via Alvaro del Portillo n. 21, 00128 Rome, Italy

**Keywords:** ethyl levulinate, levalunic acid, water removal, numerical study, pervaporation membrane reactor, operating conditions

## Abstract

A detailed numerical study of ethyl levulinate (EtLA) production with levulinic acid (LA) and ethanol (Et) in a multi-bed traditional reactor (MB-TR) and a silica-based and polymeric multi-bed pervaporation membrane reactors (MB-PVMR) was conducted and the efficiency of each design was studied under different operation conditions. Due to water production in the EtLA production process, water removal by a pervaporation system may improve process performance. Our results showed that MB-PVMR had higher performance compared with MB-TR. In addition, the silica membrane was more effective in water removal compared with the polymeric membrane. Therefore, higher LA conversion was achievable by a silica-based multi-bed pervaporation membrane reactor (SMB-PVMR). All the results were evaluated for percentage of water removal and LA conversion, based on variations in the Et/LA molar ratio, feed molar flow, reaction zone temperature, and catalyst loading. The results showed that water removal was higher than 95% and LA conversion of about 95% was attained by SMB-PVMR.

## 1. Introduction

Due to global concerns about the environment, a huge number of studies have been conducted to reduce parameters related to the environmental crisis [1]. A main source of these environmental problems is fossil fuel consumption and its cumulative amounts of pollutant emissions to the atmosphere [2,3,4]. Future energy supply and chemical production have been very intensive research fields in recent years due to the growing environmental problem and depletion of fossil fuel reservoirs. Levulinic acid is an important candidate for the production of fuel oxygenates, such as ethyl levulinate (EtLA) [5] and chemicals used in the flavoring and fragrance industries [6]. In addition, the development of new technologies to produce liquid transportation fuels from bio-based feed-stocks is important. Levulinic acid (LA) is a product formed via the fractionation of lignocellulosic biomass and cellulose; it can be obtained by hydrolysis of cellulose in the presence of acid catalysts. The main benefit of this method is that lignocellulosic biomass is abundant and inexpensive [7,8,9]. Levulinic acid esterification (LA-ESR) with ethanol can be used according to the following acid-catalyzed reaction for EtLA production:

Levalunic acid (LA) + Ethanol (Et) = Ethyl levulinate (EtLA) + water(1)

Ethyl levulinate has been proposed to be used in fuel blends. It has been demonstrated that up to 20% ethyl levulinate can be added into a mixture of 79% diesel and 1% co-additive to improve the combustion of diesel fuels [9]. In addition, ethyl levulinate is sulfur-free, thus reducing diesel emissions. A lower sulfur level in the fuel improves its lubricity, causing less wear in the engine and prolonging the life of engine components. In Europe, the lubricity of the fuel is measured with the high frequency reciprocating rig (HFRR) test, in which the addition of 20% ethyl levulinate into No. 2 base fuel was able to improve the HFRR value from 410 to 275 [10]. Biodiesel composed of fatty acid methyl esters (FAME) does not have very good cold properties and suffers from gum formation [9]. The addition of ethyl levulinate into FAME improves both features of biodiesel, which leads to a more competitive product on the fuel market. Finding a promising method for EtLA production is a critical step for using EtLA as a fuel or commercial fuel blend. Traditionally, studies were conducted in conventional reactors; however, new reactors with a more efficient design is required for reducing EtLA production costs. Water removal from the reaction zone can increase EtLA production, which was normally evaporated in traditional reactors. The membrane separation process has been used in many chemical production processes [10,11,12,13,14,15]. Water selective membranes can efficiently separate water from the reaction mixture and improve performance. In this study, multi-bed pervaporation membrane reactors (MB-PVMR) were used for ethyl levulinate production. As shown in Equation (1), the reaction of levalunic acid and ethanol in a catalytic reactor produces ethyl levulinate and water. Based on Le Chatelier’s principle, water removal from the reaction zone can lead to higher EtLA production. On the other hand, pervaporation of water from the reaction zone can lead to a little pervaporation of EtLA, which can decrease the reactor performance. Therefore, water selective membrane reactors can prevent EtLA pervaporation and simultaneously provide high water removal. In this study, a CFD-based model was developed to simulate the performance of a MB-PVMR, including silica-based polymeric membranes, compared with a MB-TR. The lower hydrothermal stability is a significant limitation of silica membranes. However, in literatures [16,17,18], some strategies were proposed for improving silica hydrothermal stability.

## 2. Developing the CFD Model

**Figure 1:**
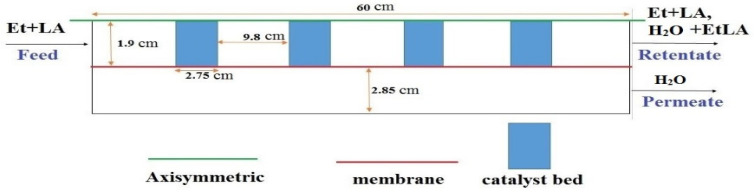
Scheme of the simulated MB-PVMR.

To simulate the performance of different reactors, a two-dimensional and axisymmetric geometry was considered. Figure 1 depicts a simple scheme of MB-PVMR performance for EtLA production with the Smopex-101 catalyst during a LA-ESR reaction, where the tested reactor includes four catalytic beds. The main assumptions of this CFD model included:

- Isothermal condition;
- Steady state;
- Negligible film transport resistance at the interface of feed/membrane;
- Constant performance of membrane and catalyst without any deactivation or concentration polarization.

### 2.1. Governing Physical Equations

The governing equations in non-porous and porous zones are given in Table 1. The source term *S_i_* in the porous momentum equation is expressed as [19]:

(2) $$S=-\frac{\mu }{K_{br}}\;u+\beta_{F}\left|u\right|u$$

where μ is the liquid mixture viscosity, *K_br_* is permeability, and $\beta_{F}$ is the Forchheimer coefficient for packed bed particles. *K_br_* and $\beta_{F}$ are defined as:

(3) $$K_{br}=\frac{d_{cat}^{2}\;\varepsilon^{3}}{180{\left(1-\varepsilon \right)}^{2}}$$

(4) $$\beta_{F}=\frac{1\cdot 75\rho }{\sqrt{150K_{br}\varepsilon^{3}}}$$

Effective diffusion in porous media, $\;D_{ei},$ depends on the structure of the porous material and the phases involved. In saturated or partially saturated porous media, the effective diffusivity is defined as:

(5) $$D_{ei}=\frac{\varepsilon }{\tau }D_{Li}$$

The fluid tortuosity for the Millington and Quirk model is:

(6) $$\tau =\varepsilon^{-1/3}$$

where ε,ρ, *d_cat_*, and $D_{Li}$ are the porosity, density of fluid, diameter of catalyst particle, and single phase diffusion coefficients for the species in fluid, respectively. The porosity and particle diameter was set to 0.3124 and 0.01 mm, respectively. Porosity for the non-porous zone was set to 1. The flow pattern of the non-porous zone momentum equation was laminar flow. *S_i_* in the porous species equation was the sink/source term of component *i*, which accounted for the addition or removal of the component *i* into the system for permeation through the membrane. In this work, as only water permeated from the retentate to the permeate side, this term appears as a sink term in the retentate side and a source term in permeate side. In other words, *Si* = 0 for all components except for H_2_O, which is calculated as:

(7) $$S_{W}=\frac{A\;J_{w}M_{w}}{V}$$

where *A* is the membrane surface, *V* the computational cell volume, *M_w_* the water molar weight, and *J_w_* is the water permeating flux across the silica-based and polymeric membrane.

Water diffusion across the Pervap2201 PMB-PVMR (polymeric multi-bed pervaporation membrane reactor) [7] can be specified with the following equation:

(8) $$J_{w}=1\cdot 19\times 10^{7}\exp\left(-\frac{E}{RT}\right)\;\left(\exp\left(2\cdot 17w_{w}\right)-1\right)\;E=49.96\;\mathrm{kJ}/\mathrm{mol}\;$$

where *w_w_* is the water mass fraction and *J_w_*, *E*, *R*, and *T* are water diffusion rates across the membrane, activation energy, gas constant, and temperature.

The mathematical model for predicting the water diffusion rate across the SMB-PVMR can be shown as [8]:

(9) $$Q_{\mathrm{memb},w}=3\cdot 278\times 10^{-11}\exp\left(18\cdot 64x_{w}\right)\exp\left(-\frac{50,377x_{w}-32,326}{RT}\right)\;\mathrm{mol}/s.m^{2}\;\mathrm{Pa}$$

(10) $$J_{w}=Q_{\mathrm{memb},w}\times \;(x_{w}\gamma_{w}P_{w}^{sat}-y_{w}P_{perm})$$

*x_w_*, $P_{w}^{sat}$, $\gamma_{w}$, $y_{w}$, and $P_{perm}$ are the mole fraction of water at the reaction zone, vapor pressure of water, activity coefficient of water, mole fraction of water in the permeate zone, and pressure of the permeate zone, respectively.

### 2.2. Chemical Kinetic Reactions

The empirical reaction rate of the LA-ESR reaction, conducted by Russo et al. [9] using a Smopex-101 heterogeneous catalyst versus the self-catalyzed form of this reaction is shown as follows:

(11) $$R_{1}=k_{1.0}c_{LA}\left(c_{LA}c_{Et}-\frac{1}{K}c_{EtLA}c_{w}\right)\;(\mathrm{self}\text{-}\mathrm{catalyzed})\;$$

(12) $$R_{2}=k_{2.0}\rho_{cat}\left(c_{LA}c_{Et}-\frac{1}{K}c_{EtLA}c_{w}\right)\;(\mathrm{heterogeneous}\;\mathrm{catalyst}=\mathrm{Smopex}\text{-}101)$$

(13) $$R_{t}=R_{1}+R_{2}\;r_{LA}=r_{Et}=-Rt\;\&\;r_{LAEt}=r_{H_{2}O}=Rt$$

where *R*_2_ and *R*_1_ are the reaction rate at the defined catalytic and non-catalytic bed, respectively. Moreover, rate constants are correspondingly defined in Equation (14).

(14) $$k_{i}=k_{i0}\;\exp\left(-\frac{E_{i}}{R}\left(\frac{1}{T}-\frac{1}{T_{ref}}\right)\right)$$

In Equation (12), $\rho_{cat}$ is the catalyst density and *K* is the equilibrium constant expressed as:

(15) $$K=K_{ref}\;\exp\left(-\frac{\Delta H}{R}\left(\frac{1}{T}-\frac{1}{T_{ref}}\right)\right)$$

The parameters used in reaction rate constants are reported in Table 2.

### 2.3. Chemical–Physical Properties

The dependence of the liquid density on the temperature for each component was evaluated according to the empirical expression [9]:

(16) $$\rho_{i}=MW_{i}\cdot \frac{A_{i}}{B_{i}^{\left[1+{\left(1-\frac{T}{C_{i}}\right)}^{D_{i}}\right]}}$$

The coefficients of Equation (16) for each component are reported in Table 3.

The calculation of the diffusion coefficient (*Di*) of the various components was carried out using the Wilke–Chang equation for liquid systems:

(17) $$D_{i}=\frac{7\cdot 4\times 10^{-8}{\left(\varphi M_{i}\right)}^{1/2}T}{\mu_{mix}V_{i}^{0.6}}$$

where *V_i_* represents the molar volume at normal boiling point (cm^3^/mol), calculated through the inverse of Equation (16) and *μ_mix_* is the viscosity of the reaction mixture (cP). The term *ϕ* is defined as the association factor and depends on the nature of the chemical components (*ϕ*_LA_ = *ϕ*_EtLA_ = 1.0, *ϕ*_Et_ = 1.5 and *ϕ*_H2O_ = 2.6). The $\varphi M_{i}$ was therefore obtained for each component according to Equation (18):

(18) $$\varphi M_{i}=\overset{n-1}{\underset{j}{\sum }}x_{i}^{j}\cdot \varphi_{i}\cdot MW_{i}\;\left(n=4;j\neq i\right)$$

where $x_{i}^{j}$ is a molar fraction; in each step, it was determined for three components, using the *i*-th component and varying the j factor [20]. The temperature dependence of viscosity was evaluated for each component in the reaction system using the empirical expression retrieved from the CHEMCAD database [20].

(19) $$\mu_{i}=\exp\left(A_{\mu i}+\frac{B_{\mu i}}{T}+C_{\mu i}\cdot \ln\left(T\right)+D_{\mu i}T^{E_{\mu i}}\right)$$

The coefficients of Equation (19) for each component are reported in Table 4.

### 2.4. Boundary Conditions and Post-Processing Definitions

In Table 5, the boundary conditions for retentate and permeate sides are presented. The following correlations were defined to describe MB-PVMR performance in the LA-ESR reaction:

(20) $$\mathrm{LA}\text{-}\mathrm{conversion}\;(\%)=\;\frac{{\mathrm{LA}}_{\mathrm{in}}-{\mathrm{LA}}_{\mathrm{out}}}{{\mathrm{LA}}_{\mathrm{in}}}\times 100$$

(21) $$\mathrm{Water}\;\mathrm{removal}\;(\%)=\frac{H_{2}O_{\mathrm{permeate}}}{H_{2}O_{\mathrm{permeate}}+H_{2}O_{\mathrm{retentate}}}\times 100$$

where LA_in_ and LA_out_ show the inlet and outlet levulinic acid molar flow rates, respectively, and H_2_O_retentate_ and H_2_O_permeate_ are the water molar flow rates in the retentate and permeate flow, respectively.

### 2.5. Numerical Method

Numerical simulations were performed using the commercial CFD package COMSOL Multiphysics 5.4 and the finite element method was used to solve the governing equations in the two-dimensional CFD model for the present work. Moreover, pressure-velocity correction was done using the coupling algorithm. Meanwhile, the equation solution was considered to be achieved when the residuals converged to values less than the magnitude of 10^−5^ and all the variable values were not changed with iteration.

### 2.6. Mesh Independency

**Figure 2:**
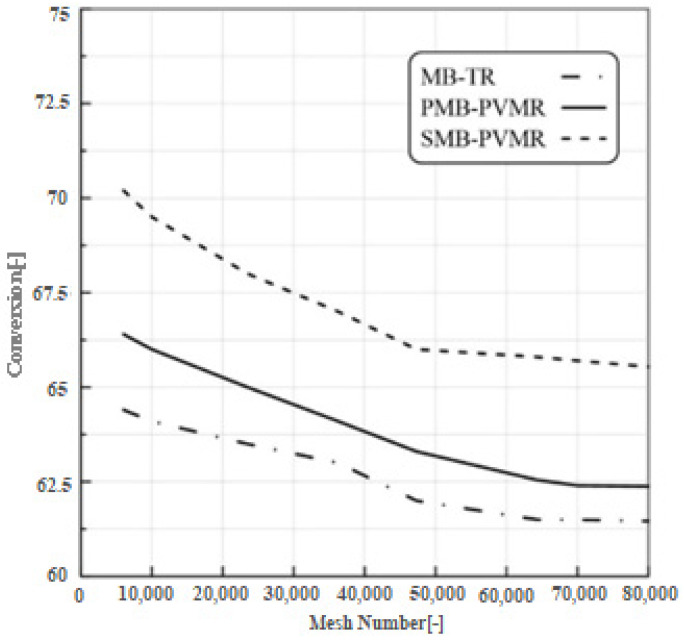
Effect of mesh number on the calculated LA conversion by CFD model (ET/LA: 1, feed flow rate 10 mm^3^/s, reaction temperature 333 K, and W = 8.6 g).

Another objective of the preliminary CFD simulations was to carry out the mesh size independency test. For this purpose, CFD simulations were carried out using different grid densities to find out the grid density beyond which the results became grid-independent. The investigated mesh numbers were 6020, 23,478, 36,240, 64,256, and 80,000. These simulations were carried out for MB-PVMR and MB-TR configurations during the LA-ESR reaction (reaction temperature 333 K, feed flow rate 10 mm^3^/s, and molar ratio of Et/LA: 1 and W = 8.6 g). The results for the grid independency tests are shown in Figure 2. The tracked parameter was the LA conversion. The results showed that, for mesh numbers higher than 48,000, the conversion did not change much with further increases in the grid numbers. Therefore, a finer grid was identified as the grid density at which the solution becomes grid-independent. This grid number (48,000 meshes) was considered in all simulations discussed in the following sections.

## 3. Results and Discussion

### 3.1. Model Validation

**Figure 3:**
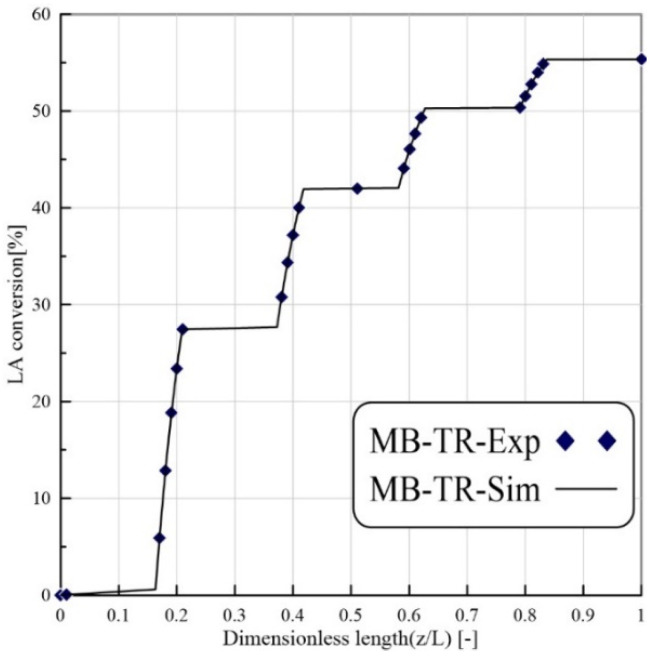
Comparison numerical results of EtLA production with experimental results achieved in Russo et al. [9] studies. Feed flow rate: 16.7 mm^3^/s, temperature: 333 K, and Et/LA = 1.

The accuracy of the CFD model was confirmed utilizing the experimental results of Russo et al. [9]. Figure 3 shows the LA conversion versus reactor length for the MB-TR during the levulinic acid esterification reaction. The operating conditions for the TR model were the same as those used in Russo et al. [11] (T = 333 K, Et/LA = 1:1, flow rate = 16.7 mm^3^/s, and W = 8.6 g). The simulation results and experimental data were consistent.

### 3.2. Evaluation of the Operating Parameter Effects

As reported in Table 6, after the preliminary CFD analysis, simulations were carried out to understand the effects of the operating parameters on the performance of the dense MB-PVMR, in terms of LA conversion and water removal during the LA ESR reaction. Indeed, this table reports the operating conditions used during the CFD modeling of the LA-ESR reaction.

The simulation sets could be divided into four parts, for which reaction temperature, catalyst loading, feed molar ratio, and feed flow rate values were changed for MB-PVMR and MB-TR.

### 3.3. LA Conversion versus Different Operating Conditions

Conversion is an important parameter that can show the process performance. It had higher importance in this study because there was only a single reaction and the reactants totally converted to the desirable product. LA conversion is dependent on different variables including feed molar ratio, feed flow rate, catalyst loading, and temperature.

#### 3.3.1. Feed Molar Ratio Effect

**Figure 4:**
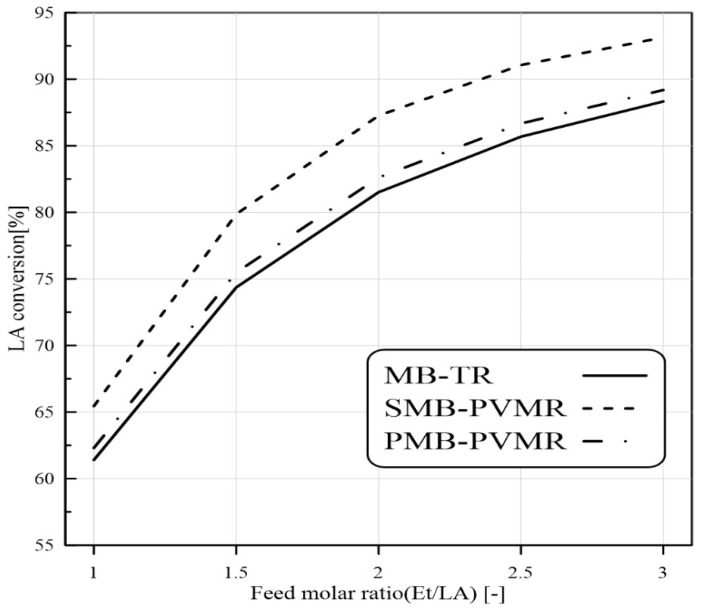
Comparison of LA conversion in MB-TR, PMB-PVMR, and SMB-PVMR based on variations in the feed molar ratio Et/LA. Feed flow rate: 10 mm^3^/s, temperature: 333 K, and W = 8.6 g.

As the first effective variable in LA conversion, the Et/LA molar ratio in the feed was studied. More contact between the Et and LA led to greater conversion of LA that was dependent on the Et/LA ratio. A greater Et/LA ratio means the LA becomes the limiting reactant. Therefore, if the other parameters remain constant, increasing the Et/LA molar ratio results in higher LA conversions. It is conventional that the reactant with the lower price should be the excess reactant in a reaction to push the process so that the expensive reactant is completely consumed. On the other hand, high amounts of Et in the feed can reduce the EtLA concentration in the product stream (because some Et will be in excess and present in the product stream without reactions). This means that more separation processes are needed and the final price of EtLA will be increased. Figure 4 shows that a higher feed molar ratio of Et/LA increases LA conversion in all cases. In addition, SMB-PVMR has higher performance compared with MB-TR and PMB-PVMR. This shows that silica membrane has a higher rate of water removal from the reaction zone.

#### 3.3.2. Feed Flow Rate Effect

**Figure 5:**
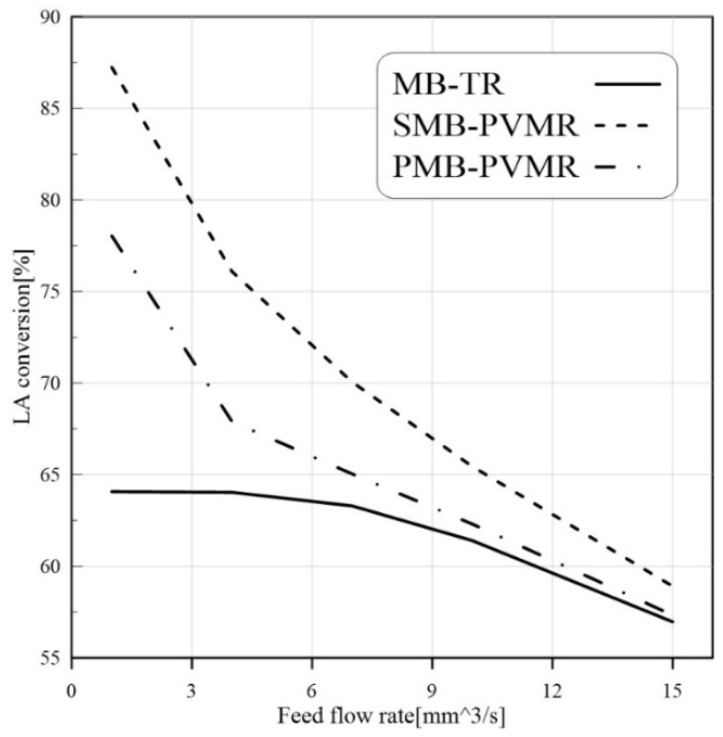
Comparison of LA conversion in MB-TR, PMB-PVMR, and SMB-PVMR based on variations in the feed flow rate. Et/LA: 1, temperature: 333 K, and W = 8.6 g.

Feed flow rate is another effective parameter that can change reactant conversions. Higher flow rates reduce the resident time of reactants in the reaction zone. Therefore, it is expected that LA conversion is reduced by enhancing the feed flow rate. As shown in Figure 5, increasing feed flow rate leads to lower conversion of LA in all reactors, but SMB-PVMR has a higher LA conversion compared with other types of reactors.

#### 3.3.3. Catalyst Loading Effect

**Figure 6:**
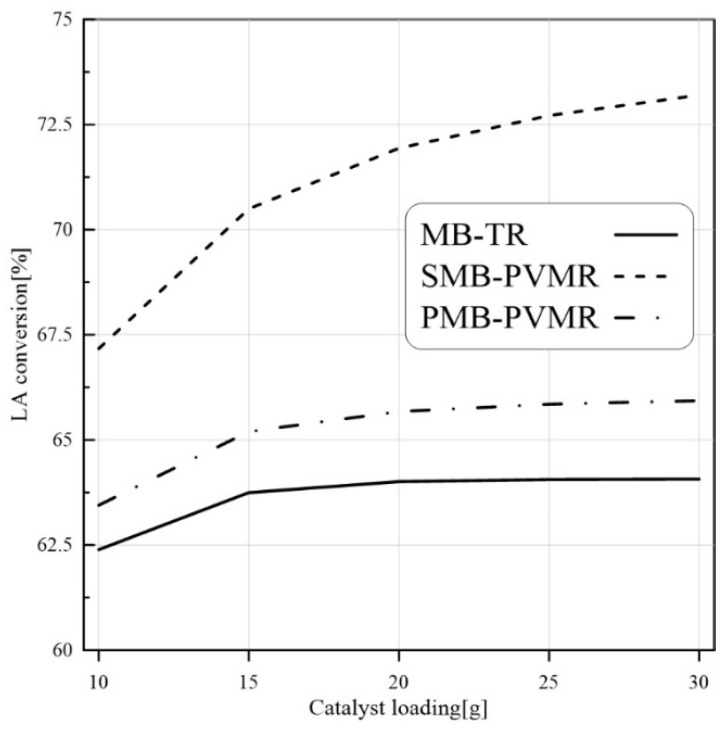
Comparison of LA conversion in MB-TR, PMB-PVMR, and SMB-PVMR based on variations in catalyst loading. Et/LA: 1, temperature: 333 K, and feed flow rate: 10 mm^3^/s.

The conversion of LA to EtLA is a catalyzed reaction, so LA conversion can be enhanced by higher catalyst loading. As illustrated in Figure 6, the numerical studies show that catalyst load enhancement increased LA conversion. In all cases, MB-TR had the lowest conversion between the three reactor types and the highest conversion was observed in SMB-PVMR.

#### 3.3.4. Temperature Effect

**Figure 7:**
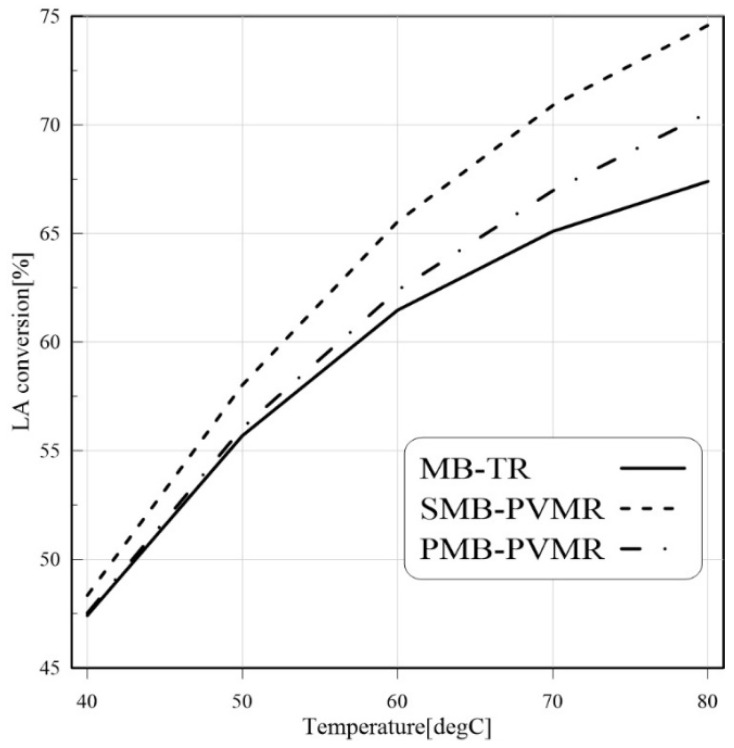
Comparison of LA conversion in MB-TR, PMB-PVMR, and SMB-PVMR based on variations in temperature. Et/LA: 1 and feed flow rate: 10 mm^3^/s and W: 8.6 g.

The EtLA production reaction is an endothermic reaction, so it is expected that temperature enhancement would lead to higher conversion of LA. As shown in Figure 7, temperature enhancement increased the LA conversion in all studied cases. Similar to previous findings, the silica membrane showed better performance compared with the polymeric membrane. In addition, the water removal capability in MRs led to higher conversion in MRs than MB-TR.

### 3.4. Water Removal at Different Operation Condition

**Figure 8:**
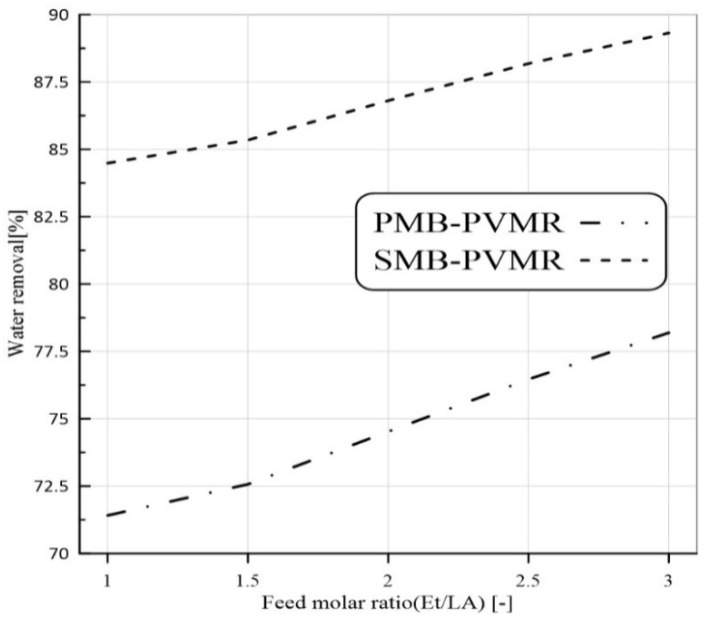
Comparison of water removal in MB-TR, PMB-PVMR, and SMB-PVMR based on variations in the feed molar ratio, Et/LA. Feed flow rate: 10 mm^3^/s, temperature: 333 K, and W = 8.6 g.

**Figure 9:**
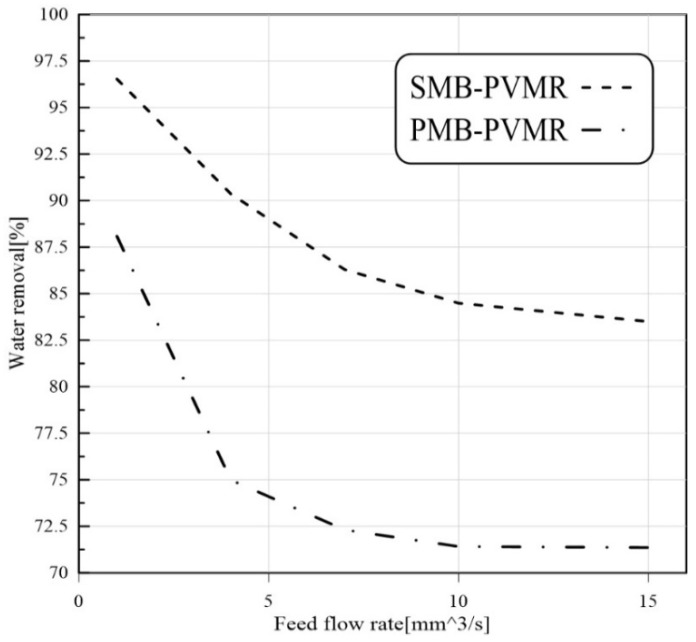
Comparison of water removal in MB-TR, PMB-PVMR, and SMB-PVMR based on variations in feed flow rate. Et/LA: 1, temperature: 333 K, and W = 8.6 g.

**Figure 10:**
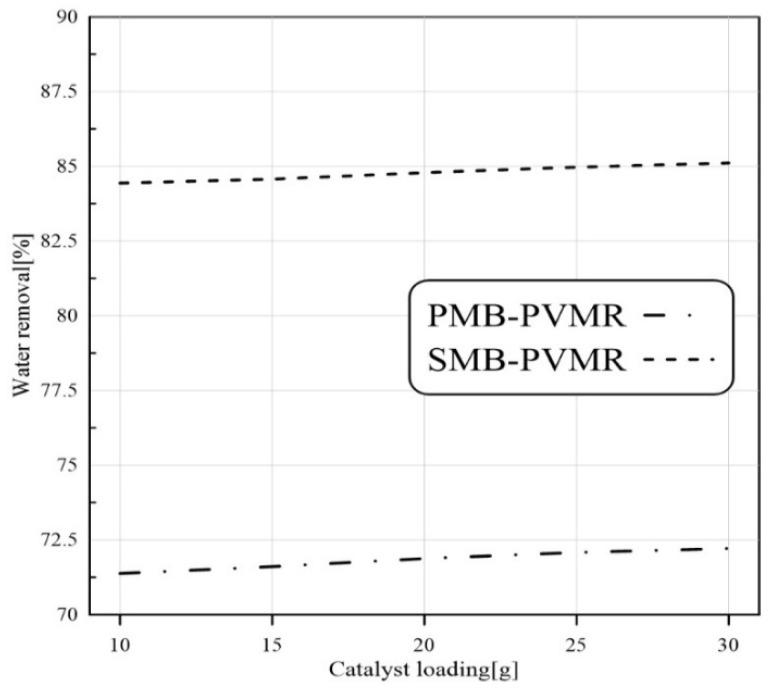
Comparison of water removal in MB-TR, PMB-PVMR, and SMB-PVMR based on the variations in catalyst loading. Et/LA: 1, temperature: 333 K, and feed flow rate: 10 mm^3^/s.

**Figure 11:**
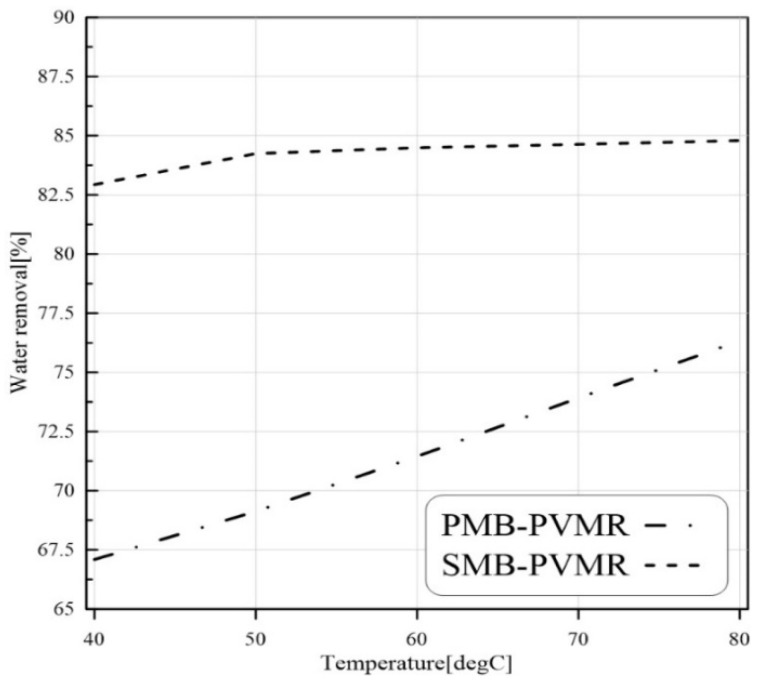
Comparison of water removal in MB-TR, PMB-PVMR, and SMB-PVMR based on variations in temperature. Et/LA: 1, feed flow rate: 10 mm^3^/s, and W: 8.6 g.

Figure 8, Figure 9, Figure 10 and Figure 11 show water removal from the EtLA reaction zone in different conditions of the feed molar ratio, feed flow rate, catalyst loading, and reaction temperature. As shown in Figure 8, increasing the Et/LA molar ratio leads to a higher percent of water removal. Higher performance of SMB-PVMR is shown in this figure. A high value of water removal (about 90%) from the reaction zone was achieved by SMB-PVMR. In all the studied feed molar ratios of Et/LA (Et/LA = 1−3), the water removal percentage of PMB-PVMR was about 10% lower than that of SMB-PVMR. Feed flow rate was another effective parameter that could change the percentage of water removal. As seen in the previous section, higher feed flow rates led to lower LA conversion; hence, the water concentration, as a product, is reduced in the reaction zone. Consequently, the driving force of water permeation from the membrane weakens and reduces the percentage of water removal, as shown in Figure 9. Enhancing catalyst loading can reduce the concentration of unreacted feeds, leading to higher water concentration in the reaction zone. Therefore, water permeate flux will be increased and there will be a higher percentage of removal, as illustrated in Figure 10. Reaction zone temperature can increase feed conversion and water concentration in the product stream. Therefore, a higher percentage of water removal is achieved, as shown in Figure 11. In addition, the temperature dependency of the water removal in PBM-PVMR is higher, meaning that the increase in the percentage of water removal due to temperature increases is more significant in PBM-PVMR than in SMB-PVMR. Overall, SMB-PVMR had a higher permeation flux and higher performance in water removal compared with PMB-PVMR; both had higher effectivity in the conversion of LA and Et to EtLA and water.

## 4. Conclusions

In this study, we investigated EtLA production in three different reactors, MB-TR, PMB-PVMR, and SMB-PVMR. Water is a product of the LA and Et reaction; thus, the EtLA production rate could be improved by water removal from the reaction zone. In this study, polymeric and silica-based PVMR were compared, and results showed PVMR performed better than MB-TR. Numerical study results showed that the Et/LA molar ratio in the feed stream could increase LA conversion and percentage of water removal. In addition, a reduction in water removal and LA conversion was found by enhancing the feed flow rate. Increases in catalyst loading and temperature pushed the process to increase water removal and LA conversion.

## Figures and Tables

**Table 1:** Governing equations in the CFD model.

Retentate	Permeate
Continuity equation ∇·(ρ·u·ε)=0	Continuity equation ∇·(ρ·u)=0
Momentum equation $\begin{array}{ll} \frac{\rho }{\varepsilon^{2}}\left(\left(u\cdot \nabla \right)\frac{u}{\varepsilon }\right)= & \nabla \cdot [-pI+\frac{\mu }{\varepsilon }\left(\nabla u+{\left(\nabla u\right)}^{\vartheta_{r}}\right)\\ & -\frac{2\mu }{3\varepsilon }\left(\nabla u\right)I]+S \end{array}$	Momentum equation $\begin{array}{ll} \rho \left(u\cdot \nabla \right)u=\nabla \cdot [ & -p_{r}I\\ & +\mu \left(\nabla u+{\left(\nabla u\right)}^{\vartheta_{r}}\right)] \end{array}$
Species equation $u\cdot \nabla c_{i}=\nabla \cdot \left[\left(D_{ei}\right)\nabla c_{i}\right]+R_{i}+S_{i}$	Species equation $\nabla \cdot \left(-D_{i}\nabla c_{i}+uc_{i}\right)=0$

**Table 2:** Values of rate constant parameters used in simulation for LA-ESR reaction [9].

Parameter	Name	Value	Units
Δ*H*	Enthalpy	15.14	kJ/mol
*E*_1_	Activation energy	32.51	kJ/mol
*E*_2_	Activation energy	39.38	kJ/mol
*K_ref_*	Equilibrium cst	3.18	-
*k*_1,0_	Rate constant	5.74 × 10^−14^	(m^3^/mol)^2^/s
*k*_2,0_	Rate constant	5.32 × 10^−10^	(m^3^/mol)·(m^3^/kg)/s
*T_ref_*	Temperature ref	333	K

**Table 3:** Numerical values of the coefficients used to calculate the density of each component.

Coefficients	LA	Et	EtLA	H_2_O
*A_i_*	0.754	1.65	0.528	5.46
*B_i_*	0.258	0.276	0.246	0.305
*C_i_*	738	514	666	647
*D_i_*	0.220	0.233	0.286	0.081

**Table 4:** Numerical values of the coefficients used to calculate the viscosity of each component, dependent on temperature [17].

Coefficients	LA	Et	EtLA	H_2_O
*A_µi_*	−12.873	7.874	−1.3913	−51.964
*B_µi_*	2295.7	781.98	1034.8	3670.6
*C_µi_*	−0.043631	−3.0418	−1.4837	5.7331
*D_µi_*	0	0	0	−5.3495 × 10^−29^
*E_µi_*	0	0	0	10

**Table 5:** Boundary conditions on the retentate and permeate sides.

Permeate Side	Retentate Side	Position
-------	inflow	Z = 0
outflow	outflow	Z = L
Water flux	Water flux	r = red line
$\frac{\partial c}{\partial r}=0$	$\frac{\partial c}{\partial r}=0$	r = green line

**Table 6:** The investigated conditions for MB-PVMR and MB-TR during LA-ESR reaction.

Operating Parameters	Temperature Effect	Feed Flow Rate Effect	Et/LA Molar Ratio	Catalyst Loading
Temperature (°C)	40–80	333	333	333
Feed flow rate (mm^3^/s)	10	1–15	10	10
Et/LA	1	1	1–3	1
Catalyst loading (g)	8.6	8.6	8.6	10–30

## Data Availability

Not applicable.
